# WINROP Identifies Severe Retinopathy of Prematurity at an Early Stage in a Nation-Based Cohort of Extremely Preterm Infants

**DOI:** 10.1371/journal.pone.0073256

**Published:** 2013-09-12

**Authors:** Pia Lundgren, Elisabeth Stoltz Sjöström, Magnus Domellöf, Karin Källen, Gerd Holmström, Anna-Lena Hård, Lois E. Smith, Chatarina Löfqvist, Ann Hellström

**Affiliations:** 1 Institute of Neuroscience and Physiology, Sahlgrenska Academy at University of Gothenburg, Gothenburg, Sweden; 2 Department of Clinical Sciences, Pediatrics, Umeå University, Umeå, Sweden; 3 Tornbladsinstitute, Lund University Hospital, Lund, Sweden; 4 Department of Neuroscience, Ophthalmology, Uppsala University, Uppsala, Sweden; 5 Department of Ophthalmology, Children’s Hospital Boston, Harvard Medical School, Boston, Massachusetts, United States of America; Massachusetts Eye & Ear Infirmary, Harvard Medical School, United States of America

## Abstract

**Objective:**

To evaluate the ability of a postnatal weight-gain algorithm (WINROP) to identify sight-threatening retinopathy of prematurity (ROP type 1) in a nation-based extremely preterm infant cohort.

**Methods:**

This study enrolled all 707 live-born extremely preterm (gestational age [GA] <27 weeks) infants, born 2004–2007 in Sweden; the Extremely preterm Infants in Sweden Study (EXPRESS). WINROP analysis was performed retrospectively in 407 of the infants using weekly weight gain to assess the preterm infant’s risk of developing ROP type 1 requiring treatment. GA, birthweight (BW), and weekly postnatal weight measurements were entered into WINROP. WINROP signals with an alarm to indicate if the preterm infant is at risk for ROP type 1.

**Results:**

In this extremely preterm population, WINROP correctly identified 96% (45/47) of the infants who required treatment for ROP type 1. The median time from alarm to treatment was 9 weeks (range, 4–20 weeks).

**Conclusions:**

WINROP, an online surveillance system using weekly weight gain, identified extremely preterm infants at risk for ROP type 1 requiring treatment at an early stage and with high sensitivity in a Swedish nation-based cohort.

## Introduction

A significant improvement in survival amongst the most preterm infants has been noted during the past 15 years [1]. Improved perinatal and neonatal care has lowered the limit of viability to a gestational age (GA) of 22–23 weeks in countries with high-quality neonatal intensive care units (NICU) [2].

Retinopathy of prematurity (ROP) is a potentially sight-threatening disease affecting premature infants [3]. Due to pathologic neovascularization of the retina after preterm birth, the most severe form of ROP (ROP type 1) may lead to retinal detachment and blindness if untreated [4].

To identify infants in need of treatment, repeated eye examinations must be performed until the retina is fully vascularized. ROP eye examinations are one of the more stressful and painful procedures preterm infants are subjected to, even when performed by an experienced ophthalmologist [5].

A population-based study of extremely preterm infants in Sweden (EXPRESS) with a GA <27 weeks recently reported that 20% of extremely preterm infants require treatment [6].

Poor postnatal weight gain during the first weeks of life is a strong predictor for the development of sight-threatening ROP type 1 [7-14]. The online monitoring tool, WINROP, developed in Gothenburg, Sweden, is based on longitudinal weekly weight measurements and indicates with an alarm if the preterm infant is at risk of developing sight-threatening ROP.

The WINROP monitoring tool was introduced and evaluated with satisfactory results in different preterm populations worldwide [13,15,16]. These studies have all retrospectively included both very and extremely preterm infants. The aim of this study was to retrospectively evaluate WINROP in a Swedish nation-based extremely preterm cohort.

## Study Population and Methods

### Study population

**Figure 1:**
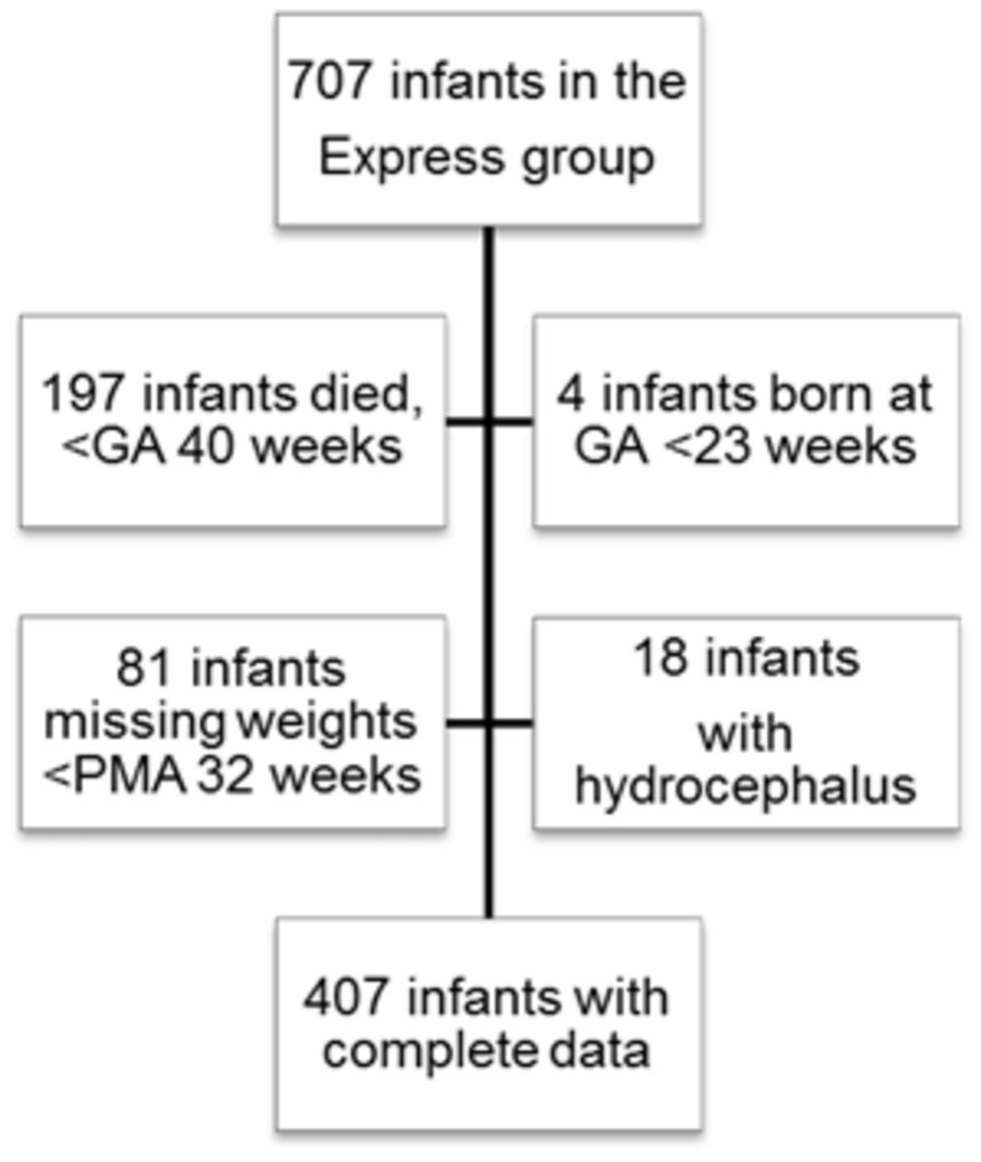
Flowchart of the study population.

In EXPRESS, all infants born in Sweden before 27 weeks of gestation between April 1, 2004 and March 31, 2007 were included. EXPRESS is a nationwide project initiated by the Swedish Association of Perinatology and the Swedish National Board of Health and Social Welfare. The aim of EXPRESS is to determine morbidity and mortality rates in extremely premature infants. Comprehensive obstetric and neonatal data were collected prospectively. GA at birth was calculated using ultrasound examinations performed before 20 weeks of gestation. Weight data of every infant throughout their hospital stay was retrieved from hospital records. Screening for ROP was separately organized and the methods, examination, techniques, and results regarding incidence, treatment, natural history, and screening have been described previously [6,17,18]. During the study period, 707 infants were born alive and these infants were included in our study (Figure 1).

WINROP use requires that the infant’s GA is from 23 to 32 gestational weeks at birth, weekly weight measurements, and physiological weight gain of <450 g/week. Infants with hydrocephalus must be excluded owing to their expected non-physiological excessive weight gain. Infants who died before 40 weeks of postmenstrual age (PMA) were excluded (n=197) as ROP grading could be incomplete in these infants. Four infants born at GA 22 weeks were excluded. Infants without weekly weight measurements up to 32 weeks PMA were excluded (n=81). Infants who were diagnosed with hydrocephalus (n=18) were excluded. Finally, 407 infants met the criteria for the WINROP analysis (Figure 1).

### ROP examination and treatment

ROP screening started during the fifth postnatal week and continued until the retina was fully vascularized or until regression of ROP [18]. The eye examinations were performed according to routine protocol, and consisted of dilated ocular fundus examinations. The results of screening examinations were entered into a study screening protocol.

For classification of ROP, the International Classification of Retinopathy of Prematurity revisited was used [19]. The recommendations of the Early Treatment for Retinopathy of Prematurity Cooperative Group were followed for treatment [20]. According to these recommendations, ROP type 1 requiring treatment is defined as any stage of ROP in zone I with plus disease; ROP stage 3 in zone I, with or without plus disease; and ROP in zone II, stage 2 or 3 with plus disease. In this study, we evaluated the ability of WINROP to predict ROP type 1.

Data regarding maximum ROP in the worst eye and eventual treatment were retrieved from the EXPRESS.

### WINROP screening

The online surveillance system WINROP uses longitudinal weekly weight gain to identify infants at risk of developing ROP type 1. Each infant’s GA, birthweight (BW), and weekly postnatal weights; from birth until an alarm is signalled or to a postmenstrual age of 35 to 36 weeks, were retrospectively entered into WINROP by a person unaware of ROP outcome.

The WINROP algorithm estimates the differences between expected safe weekly weight gain and the weight gain observed, and the values are calculated and accumulated. When the accumulated sum exceeds a limit, an alarm is signalled to indicate that the infant is at risk for severe ROP. The WINROP outcome is either no alarm or alarm. When the infant had a record of several weekly weights available, we chose to enter the weight data closest to a 7 day interval.

### Statistical analysis

The negative and positive predictive values were calculated using the sensitivity, specificity, and prevalence of ROP type 1 for the study group. 95% confidence interval (CIs) was calculated.

### Ethics Statement

The study was approved by the Regional Research Ethics Board, Lund University, Lund, Sweden. The parents provided oral informed consent for data acquisition.

## Results

### High prevalence of ROP in the extremely preterm population

In this population, 54% (219/407) were male. The median BW was 784 grams (range, 348–1315 grams), and the median GA was 25 weeks and 4 days (range 23+0/7 to 26+6/7) (Table 1). ROP was diagnosed in 69.0% (281/407) of the infants. ROP stages 1–2 developed in 39.8% (162/407). ROP stage 3, not included in ROP type 1, was diagnosed in 17.7% (72/407) and ROP type 1 in 11.5% (47/407). All infants with ROP type 1 received treatment (47/47) as well as 26.4% (19/72) of the infants with ROP stage 3 (Table 1). No infant was treated before a PMA of 32 weeks. The infants’ median PMA when receiving first treatment was 36 weeks (range, 32–47 weeks).

**Table 1:** Alarm Signal in Relation to ROP Categories and Birth Characteristics.

	**Alarm statu*s***		
	**No alarm n=88**	**Alarm n=319**	**All infants n=407**
**Birth characteristics, median (range**)			
**GA, weeks+days**	26+2/7	25+3/7	25+4/7
	(23+3/7 to 26+6/7)	(23+0/7 to 26+6/7)	(23+0/7 to 26+6/7)
**BW, g**	978 (714–1315)	740 (348–1130)	784 (348–1315)
**Sex, males n=219**	63% (56/88)	51% (163/319)	54% (219/407)
**Infants, No. (%**)	88 (21.6%)	319 (78.4%)	407
**No ROP**	38 (30.2%)	88 (69.8%)	126 (31.0%)
**ROP stages 1 & 2**	38 (23.5%)	124 (76.5%)	162 (39.8%)
**ROP stage 3**	10 (13.9%)	62 (86.1%)	72 (17.7%)
**Type 1 ROP**	2 (4.3%)	45 (95.7%)	47 (11.5%)
**Treated**	4 (6.1%)	62 (93.9%)	66 (16.2%)

Abbreviations: BW, birth weight; GA, gestational age; ROP, retinopathy of prematurity.

### WINROP identifies extremely preterm infants at risk of ROP type 1 with high sensitivity

**Figure 2:**
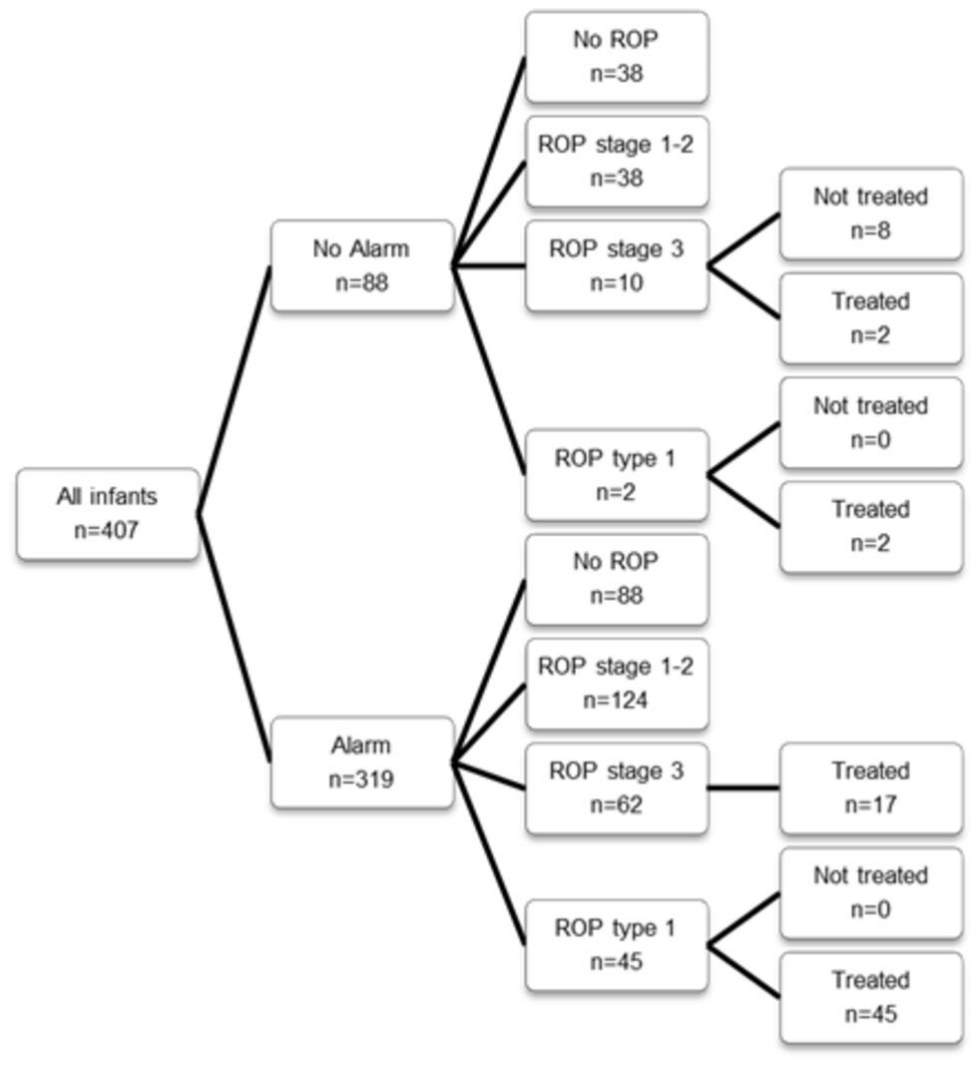
Flowchart of WINROP outcomes.

An alarm was signalled in 78.4% (319/407) of all infants and in 95.7% (45/47) of the infants with ROP type 1 (Figure 2). The specificity was 23.9% (86/360). The negative predictive was value 97.7% and the positive predictive value 14.1% (Table 2).

**Table 2:** WINROP Sensitivity, Specificity, Positive and Negative Predictive Values in Identifying Type 1 ROP.

	**Alarm status**			**% (95%CI**)	
	**Alarm**	**No Alarm**	**Total**	**Sensitivity**	**Specificity**
**ROP categories, No. of infants**					
**Type 1 ROP**	45	2	47	**95.7** (84.3-99.2)	**23.9** (19.6-28.7)
**Non-type 1 ROP**	274	86	360		
**Total**	**319**	**88**	**407**		
**Predictive value, % (95%CI**)					
**PPV**	**14.1** (10.5-18.5)				
**NPV**		**97.7** (91.2-99.6)			

Abbreviations: NPV, negative predictive value; PPV, positive predictive value; ROP, retinopathy of prematurity.

WINROP did not signal an alarm in two infants diagnosed and treated for ROP type 1. Both infants had a complicated clinical course. One had a partial bowel obstruction, long-term parenteral nutrition and antibiotic treatment, and received treatment at the PMA of 36 weeks. The second infant had severe respiratory insufficiency, with excessive oxygen treatment and intraventricular haemorrhage (IVH) grade 4. This infant was treated in only one eye for ROP type 1 at a PMA of 37 weeks. WINROP did not signal with an alarm in two infants with ROP stage 3 (ROP type 2; ROP stage 3 in zone II without plus disease) who were treated. One of these infants was only treated in one eye (Table 3).

**Table 3:** Characteristics of Infants Treated for ROP but not Signalling a WINROP Alarm.

**Infants**	**GA (weeks+days**)	**BW**	**Time at treatment & ROP type**	**Clinical Course**
**#1**	25+1/7	960g	PMA 37 weeks ROP type 1	Partial bowel obstruction and long-term parenteral nutrition.
**#2**	25+3/7	936g	PMA 38 weeks ROP type 1 One eye treated.	Severe hospital course, excessive oxygen treatment, IVH 4
**#3**	26+3/7	1010g	PMA 36 weeks ROP type 2	No severe complications
**#4**	26+4/7	910g	PMA 37 weeks ROP type 2, One eye treated	No severe complications

Abbreviations: BW, birth weight; GA, gestational age; IVH, intraventricular hemorrhage; PMA, postmenstrual age; ROP, retinopathy of prematurity

### WINROP sends an early signal when the infant is at risk of developing ROP type 1

**Figure 3:**
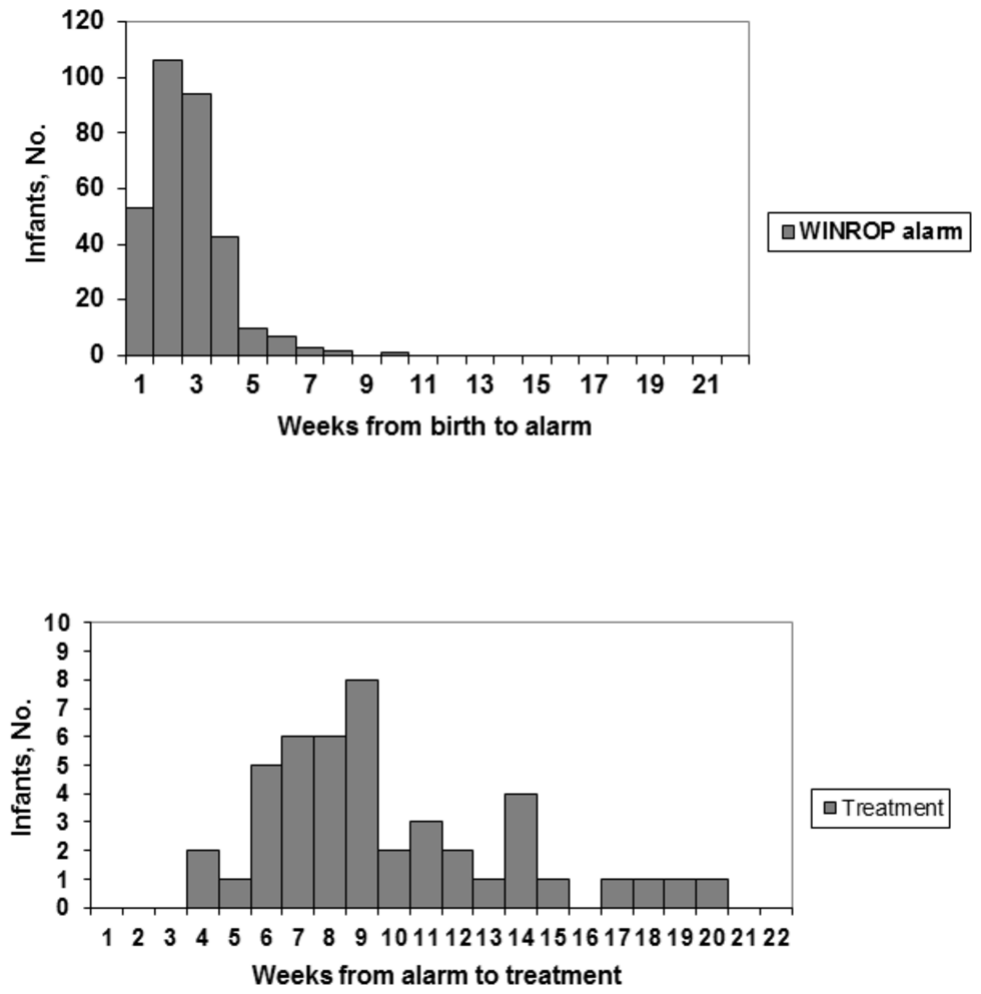
Time from birth to the WINROP alarm (A) and time from the alarm to treatment (infants with ROP type 1) (B).

The median time from birth to alarm was 3 weeks (range 1–10 weeks). The infants with ROP type 1 who received an alarm had a median time from alarm to first treatment of 9 weeks (range 4–20 weeks) (Figure 3). The infants received their alarm at least 4 weeks before their treatment.

## Discussion

In this first nation based study, WINROP identified extremely preterm infants with ROP type 1 with high sensitivity (95.7%). The extremely preterm infant is at high risk of developing ROP, but also extremely fragile. Every intervention and investigation must be carefully considered before being implemented owing to the infant’s vulnerability. The ROP eye examination is a painful and stressful procedure [21]. Changes in oxygen saturation, respiratory rate, and heart rate measured during the examination can be signs of distress [22].

Todays established screening criteria; GA and BW results in numerous unnecessary eye examinations to identify the approximately 20% of extreme preterm infants in need of treatment. Although it is crucial to correctly identify infants who require treatment, it would be beneficial if infants not in need of treatment could be spared at least some stressful examinations. In the present cohort a mean of 11 eye examinations (range 2–30) were performed/infant. The infants with no ROP and no alarm had a mean of 6 examinations/ infant. If infants with no alarm and no ROP in this cohort would have received routine ROP examination at three time points; PMA 31 weeks, PMA 33 weeks and PMA 36 weeks these infants number of examinations would have been reduced by 53% (130/244). Even though the number of examinations spared in the whole cohort is limited, for the individual infant, with no alarm and no ROP, a reduction of half of their examinations could result in an important improvement in their well-being.

Infants that develop ROP without previous alarm must, of course, depending on the severity of their ROP, be examined according to clinical judgement and present screening recommendations.

Two of the infants with ROP type 1 requiring treatment did not signal an alarm. The main characteristics of these infants were their severely complicated clinical courses. Thus, when using WINROP to complement established screening schedules, the clinical judgment regarding the infants’ risk of developing severe ROP must always supersede the WINROP outcome.

In this study the sensitivity was 95.7% similar to previously performed studies in Sweden [11,12]. The specificity of 23.9% was lower than in previously performed studies in Sweden as well as in Canada, the U.S, Brazil and Korea [11-13,16,23,24]. The reason for this finding is most likely that the present study includes a cohort of extremely premature infants, who have the poorest postnatal weight development and thus are more likely to signal an alarm in WINROP.

In this study, the WINROP alarm signalled weeks/months before the development of severe ROP, giving the neonatologist and the ophthalmologist an early prediction of the infant’s future. In addition to being a sight-threatening disease, the severity of ROP is also associated with later neurologic impairment and even death [25,26]. Severe ROP and poor early postnatal growth is a multifactorial complication in the preterm infant. Maximal nutritional support/improved weight gain and interventions for high risk infants may reduce the infants’ long term impairment associated with prematurity [27]. Other considerable risk factors for developing ROP are low IGF-1 levels [28], poor nutritional intake [27], hyperglycaemia [29,30], insulin treatment [31], corticosteroid treatment [31], insufficient intake of DHA [32] and, of course, fluctuating/high oxygen concentrations [33]. An interesting study by Pawlik et al supplementing preterm infants with omega 3 fatty acids demonstrated positive effects on ROP outcome [34]; however, no information regarding weight gain was provided in that report. In addition, there are on-going preventative studies evaluating supplementing preterm infants with DHA/EPA (EU-nr 2008-000046-31) and IGF-I (ClinicalTrials.gov Id: NCT01096784) in an attempt to improve postnatal growth and normalize retinal angiogenesis.

The strength of this study is that for the first time the WINROP surveillance system have been evaluated in a nation-based cohort. This is also the first time WINROP has been evaluated in a cohort of selectively extremely preterm infant. A limitation of this study is that the WINROP algorithm not yet have been developed for infants born at a GA of 22 weeks, consequently these infants had to be excluded from this study. Further development and validations of the WINROP algorithm have to be made before the infants born at a GA of 22 weeks can be included in the surveillance system. Another limitation in this study is the number of infants excluded, 16% (81/506), due to missing weekly weights < 32 GA weeks. These excluded infants were more immature than the participating infants; their median GA was 25 week and 1 day and their median BW was 730 g. One reason to this finding could be that more immature infants have higher morbidity and weekly weight measurement is not always possible to perform due to the infants’ severe illness. Since we have no data about the infants’ morbidity we can only speculate that this is the reason to the 16% of infants excluded due to missing weekly weight measurements.

The web-based weight-monitoring ROP screening tool, WINROP identified infants at high risk of sight-threatening ROP type 1 at an early stage in this population-based cohort of extremely preterm infants. Using WINROP to supplement traditional ROP screening programs could significantly reduce the number of stressful eye examinations for extremely preterm infants who do not signal a WINROP alarm.
